# Primary mediastinal clear cell sarcoma: a case report and review of the literature

**DOI:** 10.1186/s13000-016-0594-z

**Published:** 2017-01-13

**Authors:** Long Jin, Yuxia Sui, Haili Zhu, Zhizhong Chen, Shuguang Liu

**Affiliations:** 1 Department of Pathology, Provincial Clinical Medical College of Fujian Medical University, Fujian Provincial Hospital, Fuzhou, 350001 Fujian Province People’s Republic of China; 2 Department of Pharmacy, Provincial Clinical Medical College of Fujian Medical University, Fujian Provincial Hospital, Fuzhou, 350001 Fujian Province People’s Republic of China; 3 The State Key Laboratory of Organ Failure Research, National Clinical Research Center of Kidney Disease, Nanfang Hospital, Southern Medical University, Guangzhou, 510515 People’s Republic of China; 4 Department of Pathology, The Eighth Affiliated Hospital, Sun Yat-sen University, 3025 Shennan Middle Road, Shenzhen, 518033 Guangdong Province People’s Republic of China

**Keywords:** Clear cell sarcoma, Mediastinum, Fluorescence in situ hybridisation, EWSR1

## Abstract

**Background:**

Clear cell sarcoma (CCS) is a rare malignant soft-tissue neoplasm that displays melanocytic markers and exhibits striking histopathological features. The tumour has a predilection for the lower extremities and rarely presents in the mediastinum.

**Case presentation:**

We present a case of primary mediastinal CCS in a 57-year-old man. Computer tomography (CT) revealed a 12 × 12 × 7.5 cm mass in the anterior mediastinum. Microscopically, the tumour mainly consisted of epithelioid cells with oval vesicular nuclei and eosinophilic cytoplasm. Immunohistochemically, the tumour was positive for human melanoma black 45 (HMB-45) and vimentin but negative for S-100 and Melan-A. Fluorescence in situ hybridisation (FISH) showed a translocation involving the EWSR1 gene region.

**Conclusion:**

This report will illustrate that the mediastinum is a potential site for primary CCS and FISH plays an important role in making a conclusive diagnosis.

## Background

CCS is a rare aggressive sarcoma accounting for only 1% of soft-tissue sarcoma. It usually occurs in adolescents and young adults. The first description of a case of CCS is attributed to Enzinger in 1965, which called it a “malignant melanoma of soft parts” due to its melanocytic differentiation [1]. The tumour often arises in deep soft tissue of the lower extremities, especially the region of the foot and ankle, in association with fascia, tendons, or aponeuroses. It is rarely located in visceral sites [2–7].

Common tumours in adults that occur in the anterior mediastinum are thymomas, lymphomas, and germ cell tumours. Other soft-tissue tumours that arise in the anterior mediastinum can include leiomyosarcoma, malignant peripheral nerve sheath tumour, liposarcoma, synovial sarcoma, angiosarcoma, and extraskeletal osteosarcoma, among others. A primary CCS in the anterior mediastinum is extremely rare. The present study reports an unusual case of CCS in the mediastinum, confirmed by FISH analysis and immunohistochemical study.

## Case presentation

**Fig. 1:**
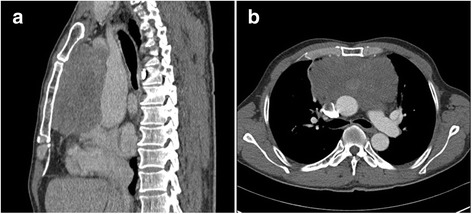
Chest computed tomography (CT). **a**, **b** The tumour mass was located in the anterior mediastinum with heterogeneous enhancement

A 57-year-old man was admitted to our hospital with complaints of a 1-month history of chest distending pain. He had no history of cutaneous malignancy. On admission, a chest CT revealed a giant lobulated mass with uneven density approximately 12 × 12 × 7.5 cm in size occupying the anterior mediastinum (Fig. 1a and b). There were no obvious calcification and fat density shadows. The mass showed a slight heterogeneous enhancement on a contrast-enhanced CT scan. The serum levels of tumour markers, namely alpha-fetoprotein (AFP) and carcinoembryonic antigen (CEA), were within normal limits. A median sternotomy was performed. A giant circumscribed and partly encapsulated mass was found attached to the right thymus. The tumour occupied the anterior mediastinum and invaded the right mediastinal pleura. Scattered 2- to 4-mm nodules were found on the surface of the pericardium and the upper lobe of the right lung.

Grossly, the mass was well-demarcated, measuring 10 × 10 × 5 cm in size and yellow-grey on the cut surface. A massive necrosis was identified.

**Fig. 2:**
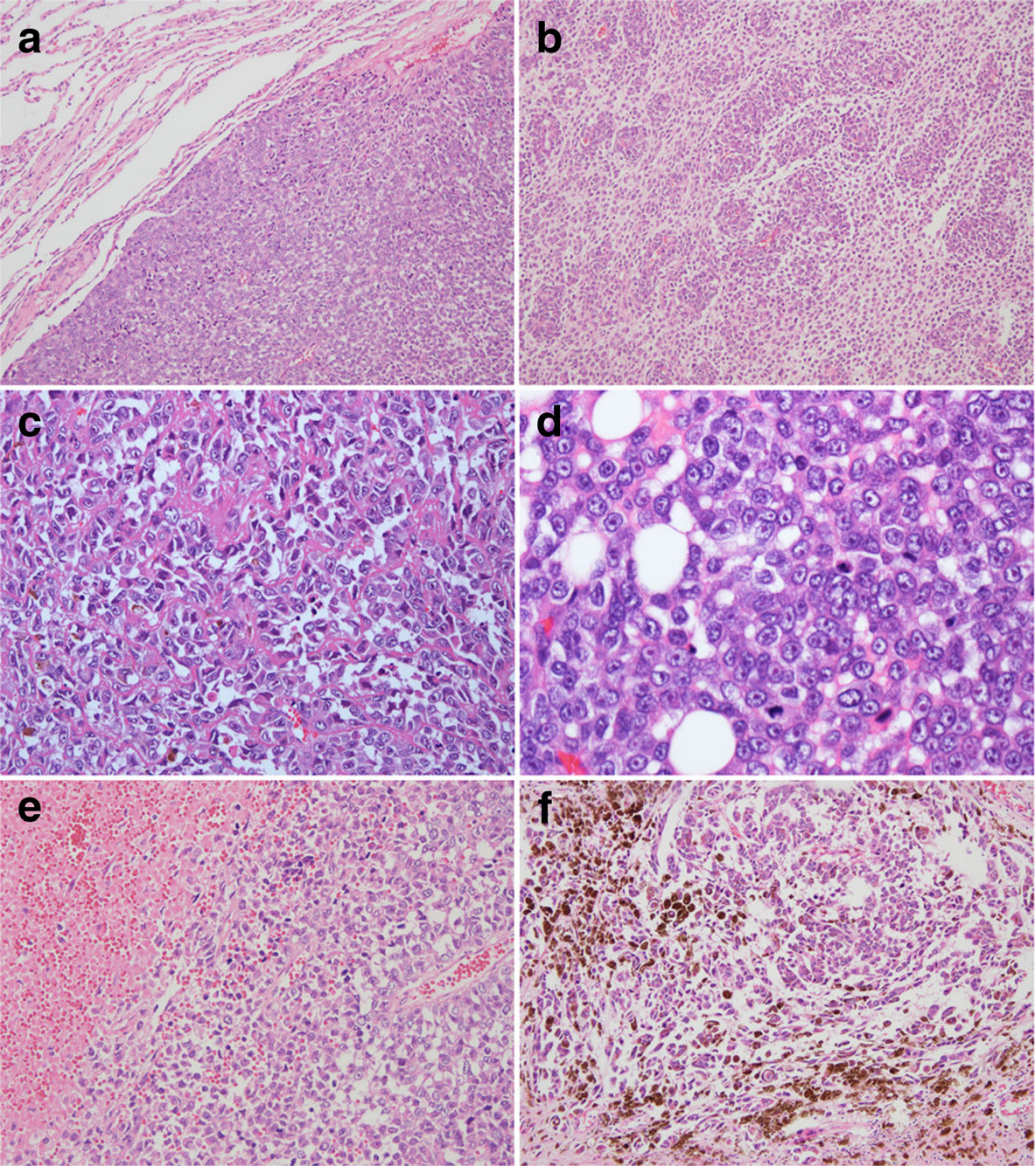
**a** The tumour demonstrated expansive growth and infiltrated into the adjacent lung tissue (magnification × 40). **b** The tumour mainly consisted of epithelioid or polygonal cells arranged in sheets (magnification × 100). **c** Clusters of tumour cells were set in a fibrous stroma (magnification × 200). **d** Tumour cells have eosinophilic cytoplasm and oval vesicular nuclei with prominent eosinophilic nucleoli (magnification × 400). **e** A large necrosis was found (magnification × 100). **f** There were some melanin-producing tumour cells (magnification × 100)

Histologically, the tumour demonstrated expansive growth and infiltrated the adjacent lung tissue (Fig. 2a). The tumour mainly consisted of epithelioid or polygonal cells arranged in sheets (Fig. 2b). Clusters of tumour cells were set in a fibrous stroma (Fig. 2c). These tumour cells had oval vesicular nuclei with prominent eosinophilic nucleoli and palely eosinophilic cytoplasm (Fig. 2d). A large necrosis was found (Fig. 2e). A proportion of the cells contained melanin (Fig. 2f).

**Fig. 3:**
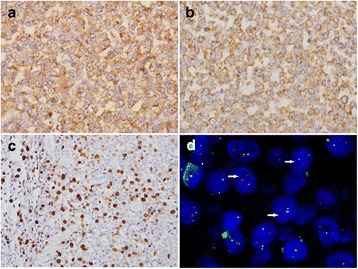
**a** Immunohistochemical examination showed positive expression of vimentin (magnification × 400). **b** Immunohistochemical examination showed positive expression of HMB45 (magnification × 400). **c** Approximately 30% of the tumour cells were positive for the proliferation marker (Ki67, magnification × 200). **d** Dual color interphase fluorescence in situ hybridisation utilizing the EWSR1 break-apart probe. Split red and green signals within a single cell demonstrated the presence of a EWSR1 rearrangement (indicated by white arrows)

Immunohistochemical studies showed a strong but diffuse distribution of the markers vimentin (Fig. 3a) and HMB-45 (Fig. 3b). These cells were negative for Melan-A, S-100, CD117, CK19, CD56, CD99, synaptophysin, chromogranin A, CD34, TTF-1, CD2, CD5, CD20, and ALKp80. The Ki-67 index was about 30% (Fig. 3c).

Dual colour interphase fluorescence in situ hybridisation utilising the EWSR1 break-apart probe was performed on a paraffin section. A clear separation of red and green signals within a single cell was identified in most cells, which demonstrated the presence of the EWSR1 rearrangement (Fig. 3d). No BRAF mutation was detected.

To date, 8 months after operation, the patient is well and without evidence of a recurrence or metastasis.

## Discussion

CCSs are a distinctive form of soft-tissue neoplasms arising from the deeper soft tissues of the extremities in 90–95% of cases. They make up 1% of all soft-tissue sarcomas and mainly affect young adults aged 20–40, with a slight female bias. CCSs also arise in other rare locations outside of the extremities, such as the kidney [8], stomach [9], and scapula [10]. In the present case, the mass was located in the anterior mediastinum.

Primary mediastinal CCSs are extremely rare. Of all published studies on CCSs, there are only 2 reported cases in the literature involving mediastinum [11, 12]. Morphologic appearances in both cases were similar to cutaneous melanoma. The first case was a 59-year-old woman with superior mediastinal involvement. The tumour was 12 × 6 × 5 cm in size and the cut surface showed a diffuse area of necrosis and cystic change. Although the tumour cells showed an absence of melanocytic differentiation, as demonstrated by the lack of expression of markers such as HMB-45 and Melan-A, FISH analysis showed EWS gene rearrangement. The second patient was a 63-year-old man with a right upper mediastinum tumour. The tumour was 8.5 × 5.5 × 5.5 cm in size. Tumour cells were positive for HMB-45 and S-100, but a gene analysis was not performed. Obviously, our case is the first case of a CCS involving the mediastinum supported by demonstration of both EWSR1 gene rearrangement and melanocytic differentiation.

Three cases of mediastinal CCSs, including our case, shared some common characteristics, such as a tumour size over 5 cm and a diffuse area of necrosis. Since the mediastinum is the internal space in the thoracic cavity between the lungs surrounded by loose connective tissue, mediastinal tumours tend to grow very large. These tumours produce few symptoms and therefore are often detected at the later stage. With the increase of tumour size, the central area of the tumour is prone to develop necrosis as a result of an insufficient blood supply.

Mediastinal CCSs also have similar morphological appearance. As indicated in our case, the tumour was composed of epithelioid cells with ovoid vesicular nuclei and eosinophilic cytoplasm. These cells infiltrated the adjacent lung tissue, showing an aggressive growth pattern. CCS is supposed to derive from neural crest cells and often shows melanocytic differentiation, which is confirmed by the expressions of S-100, HMB-45, and Melan-A. These markers are helpful for distinguishing CCSs from other types of soft tumours. In our case, the tumour cells were positive for HMB-45. However, we were unable to identify either S-100 or Melan-A in multiple sections of the tumour. Due to the inconsistent expression of melanocytic markers and its unusual site, more tumours should be rules out before making a diagnosis of CCS.

In patients without cutaneous involvement and a history of melanoma, distinguishing CCS from metastatic malignant melanoma may have diagnostic difficulties because CCS shares striking histological and immunohistochemical similarities with cutaneous melanoma. Fortunately, molecular genetic characterisation of CCS has been shown to be specific for t(12;22) translocation, which has not yet been identified in melanoma [13, 14]. Previous molecular studies showed that cutaneous melanoma has frequent BRAF mutations, but not in CCS [15, 16]. Therefore, we strengthened the diagnosis by using FISH analysis, which demonstrated an unequivocal EWSR1 gene rearrangement and lack of BRAF mutation. Other potential differential diagnoses include thymic carcinoma, lymphoma, neuroendocrine tumours, germ cell tumours, and other types of sarcomas. A careful microscopic examination and a panel of immunohistochemistry can rule out most of these diagnoses. Sometimes the distinction between the tumours may be difficult only by examining morphological features and an immunohistochemical study; therefore, a molecular genetics study is necessary for the correct diagnosis.

CCS has an aggressive behaviour. Poor prognosis of CCS is closely related to tumour size > 5 cm and the presence of necrosis, metastasis, and local recurrence. Although the patient has not shown any evidence of tumour recurrence or metastasis during the follow-up period for 8 months, the tumour’s diameter > 5 cm and massive necrosis indicated a poor prognosis.

## Conclusions

In conclusion, we report an extremely rare mediastinal CCS, a high grade of soft-tissue sarcoma with a distinct molecular gene rearrangement and with morphological features. The present case demonstrates that the mediastinum is a possible site of CCS and that a molecular genetics study is necessary for the correct diagnosis.

## Abbreviations

AFP: Alpha-fetoprotein
CCS: Clear cell sarcoma
CEA: Carcinoembryonic antigen
CT: Computer tomography
FISH: Fluorescence in situ hybridisation
HMB45: Human melanoma black 45
